# The Frequency of Catamenial Epilepsy in Female Epileptic Patients of Reproductive Age Group Presented to the Tertiary Care Hospital

**DOI:** 10.7759/cureus.11635

**Published:** 2020-11-22

**Authors:** Deepak Kumar, Samar Iltaf, Anila Umer, Meraj Fatima, Muhammad Zaheer, Kiran Waqar, Komal Girdhari

**Affiliations:** 1 Neurology, Ghulam Muhammad Mahar Medical College, Sukkur, PAK; 2 Neurology, Dow University of Health Sciences, Karachi, PAK; 3 Neurology, Jinnah Postgraduate Medical Centre, Karachi, PAK; 4 Neurology, Dow University of Health Sciences, Dow International Medical College, Karachi, PAK; 5 Neurology, Lady Reading Hospital Peshawar, Peshawar, PAK; 6 Neurology, Fazia Ruth Pfau Medical College Karachi, Karachi, PAK; 7 Internal Medicine, Ghulam Muhammad Mahar Medical College, Sukkur, PAK

**Keywords:** catamenial epilepsy, antiepileptic drugs, levetiracetam, carbamazepine, reproductive age

## Abstract

Background and aim

Catamenial epilepsy is the type of seizures during the reproductive phase of menstruation due to hormonal changes during the different phases of menstruation. This study aims to evaluate the frequency of epileptic seizures in women during the menstruation cycle and its management.

Material and methods

This study was conducted at the neurology department of Jinnah Postgraduate Medical Centre (JPMC), Karachi, Pakistan. The study's duration was six months, from the 22^nd^ of January 2020 to the 22^nd^ of July 2020. The sample size for catamenial epilepsy in female epileptic patients of reproductive age was 78%. After approval by the ethical committee of JPMC, data collection started. Data was collected and analyzed in the Statistical Package for the Social Sciences (SPSS, version 22; IBM Inc., Armonk, USA). Mean, and the standard deviation was calculated for age, duration of epilepsy, duration of antiepileptic, and antiepileptic drug. A Chi-square test was applied, and p≤0.05 was considered a statistically significant difference.

Results

A total of 184 female patients of reproductive age were selected for this study. The mean duration of epilepsy was 15.96 ± 8.85 months. The mean duration of antiepileptic drugs was 11.16 ± 7.53 months. In 73 patients (39.7%), EEG showed increased seizure activity during particular phases of the menstrual cycle. Catamenial epilepsy was seen in 73 patients (39.7%). The stratification according to age, duration of epilepsy, duration of antiepileptic drugs, the antiepileptic drug was done to observe the effect of these modifiers on catamenial epilepsy.

Conclusion

Catamenial epilepsy is relatively common epilepsy. The physician should evaluate patients when the seizures are refractory to the treatment. The females should manage a seizure diary, which will be beneficial in the management of epilepsy. In women with epilepsy, catamenial epilepsy should be considered in the diagnosis when the seizures are refractory to optimal treatment.

## Introduction

The female epileptic patients may have seizure patterns associated with hormonal changes in estrogen and progesterone levels [1]. The estrogen and progesterone levels have been shown to have marked effects on neuronal hyperexcitability [2].

Catamenial epilepsy (CE) is defined as the type of seizures related to the menstrual cycle [3]. It does not specify any seizure localization, type of seizure, or any epileptic syndrome. The exact mechanism of catamenial epilepsy is unknown. The most accepted criterion for diagnosing catamenial epilepsy is doubling a baseline seizure frequency during a specific phase of the menstrual cycle [4-7]. The reported prevalence of catamenial clustering among women with epilepsy varies widely between 10 to 78 percent. This is mainly due to methodological differences among studies, which have used different patient populations, the various criteria for diagnosis, and different recording mechanisms to link seizures with menstrual cycle phases [6, 7]. In studies that used the above definition and rigorous recording methods, approximately one-third of women with medically refractory epilepsy have a catamenial exacerbation pattern [8]. While catamenial epilepsy necessarily affects women of child-bearing age, no other demographic features or other risk factors have been clearly defined for this phenomenon. One study found that a catamenial pattern was more common in the younger half (median age 40 years) of their population of 100 women of childbearing age with intractable focal epilepsy [9]. This type of epilepsy is the common subtype occurring in approximately one-third of females with epilepsy and is defined as an increased frequency of seizures in relation to a woman's menstrual cycle [10].

It has been observed that 10% to 70% of fertile women with epilepsy have catamenial seizures. There is a lot of variability in the prevalence of catamenial epilepsy, which might be due to the different criteria used for defining seizures associated with the menstrual cycle based on patients' self-reporting and maintaining diaries and other inaccurate methods of recording seizures relating to menstruation. In the females with the anovulatory cycle, in which the luteal phase of menstruation is characterized by low levels of progesterone, the seizure frequency increases in the premenstrual phase because the estrogen level increase in their mid-cycle surge, without any significant increase in progesterone levels [11]; the imbalance in serum estrogen/progesterone ratio during the reproductive cycle bring about an increased or decreased risk of seizure occurrence. Till now, there are no specific drug treatments for catamenial epilepsy; however, non-hormonal and hormonal therapies have been proposed [12]. In one study, 59 women with catamenial epilepsy and 215 with non-catamenial epilepsy were included, 47 patients (79.7%) with catamenial epilepsy and 48 patients (22.3%) with non-catamenial epilepsy remained seizure-free throughout their pregnancy (odds ratio [OR] = 2.612, 95% confidence interval [CI] 1.901-3.323, p < 0.001), while remaining 30 (50.8%) in the CE group and 18 (8.4%) from the non-CE group showed reduction in seizure frequency during pregnancy (OR = 2.427, 95% CI 1.724-3.129, p < 0.001) [13]. Overall, the seizure-free patients were noted less than what is being reported in other published studies.Approximately 35% were seizure-free during the whole gestation period. In another large prospective study of over 3,000 pregnancies, 66.6% remained seizure-free throughout pregnancy [14].

The cyclic hormonal influence of catamenial seizure exacerbations is consistent with the neurophysiologic activity of estrogen and progesterone levels. For the women with catamenial epilepsy who have regular menstrual cycles, intermittent treatment approaches may be utilized. These interventions are mainly targeted at adding or increasing anti-seizure drugs during predictable vulnerable days for breakthrough seizures of the menstrual cycle, such as perimenstrual (C1 pattern), at ovulation (C2 pattern), and during the luteal phase (C3 pattern) [15]. As limited international and very limited local data is available on the frequency of catamenial epilepsy in female epileptic patients of reproductive age group under treatment, and there is wide variation in the prevalence of catamenial epilepsy; therefore, the present study is designed to assess the frequency of catamenial epilepsy in female epileptic patients of reproductive age group under treatment. In case of a significantly high frequency of catamenial epilepsy, we can advise a strategy of having close follow-up and increasing the dose of antiepileptics in female epileptics of the reproductive age group in order to improve the outcome [16].

## Materials and methods

This is a cross-sectional study conducted at the neurology department of Jinnah Postgraduate Medical Centre (JPMC), Karachi. The study's duration was six months, from the 22^nd^ of January 2020 to the 22^nd^ of July 2020. The sample size was 78% and was calculated based on the frequency of catamenial epilepsy in female epileptic patients of reproductive age as followed: confidence level 95%, bond on error 6%, sample size (n) = 184 for epileptic patients of reproductive age; by using this formula n= z2p (1-P) /d2 (N-1)+Z2 P) (1-P) [11].

We included patients aged 12-49 years, female gender, epileptic patients (as per operational definition) of the reproductive age group for more than six months, on anti-epileptics for more than three months. In our study, we excluded all these conditions: pregnant women, assessed by history and positive result of beta human chorionic gonadotrophin levels (HCG); chronic liver disease, assessed by history, physical examination and ultrasonographic findings (coarse echotexture of the liver, irregular margins, increased portal vein diameter > 13mm, splenomegaly); chronic renal failure, labeled based on glomerular filtration rate (GFR) < 90ml/minute; critically ill patients, admitted in high dependency unit or intensive care unit and congestive cardiac failure (ejection fraction [EF] < 25% on echocardiography). The non-probability consecutive sampling technique was applied. After approval from the ethical committee of JPMC, data collection started. Female epileptic subjects (as per operational definition) of the reproductive age group for more than six months, attending inpatient or outpatient department of neurology in JPMC, Karachi, and on anti-epileptics for more than three months, with a history of antiepileptic drugs taken, i.e., levetiracetam, phenytoin, carbamazepine. All these patients were specifically seen for catamenial epilepsy. 

All data on demography, clinical history, and BMI were recorded by a principal investigator on a pre-designed proforma. Informed written consent and permission from the ethical committee JPMC were taken before enrolment. Exclusion criteria were followed strictly to avoid confounding variables. Data was collected and analyzed in the Statistical Package for the Social Sciences (SPSS, version 22; IBM Inc., Armonk, USA). Mean, the standard deviation were calculated for age, duration of epilepsy, duration of antiepileptic drugs. Frequency and percentages were calculated for the antiepileptic drug (levetiracetam, phenytoin, carbamazepine), EEG showing increase seizure activity during particular phases of the menstrual cycle, the presence of catamenial epilepsy. Effect modifiers like age, duration of epilepsy, duration of antiepileptics, and antiepileptic drug were controlled through stratification. A Chi-square test was applied, and p≤0.05 was considered a statistically significant difference.

## Results

**Figure 1:**
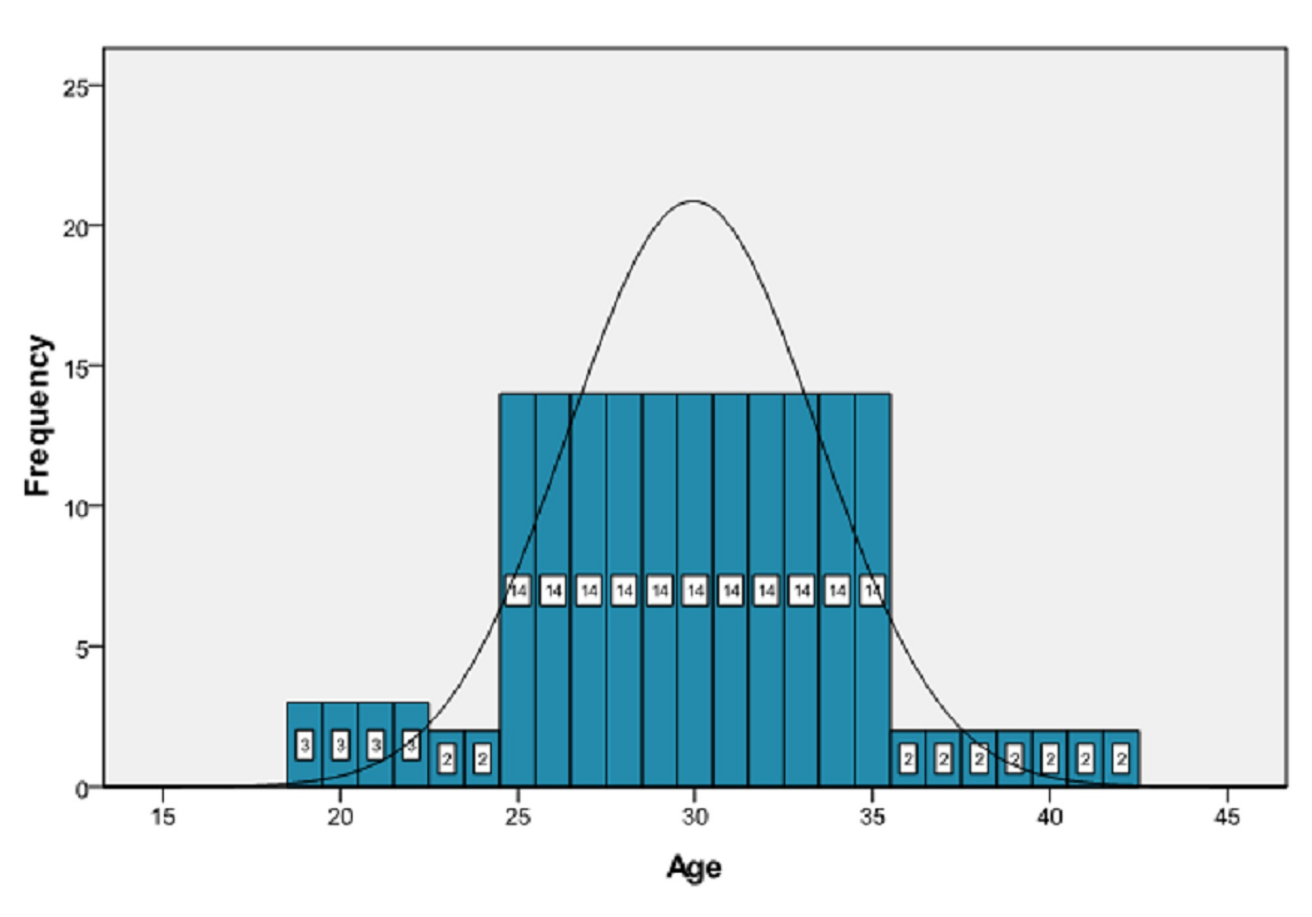
Frequency distribution of age

**Figure 2:**
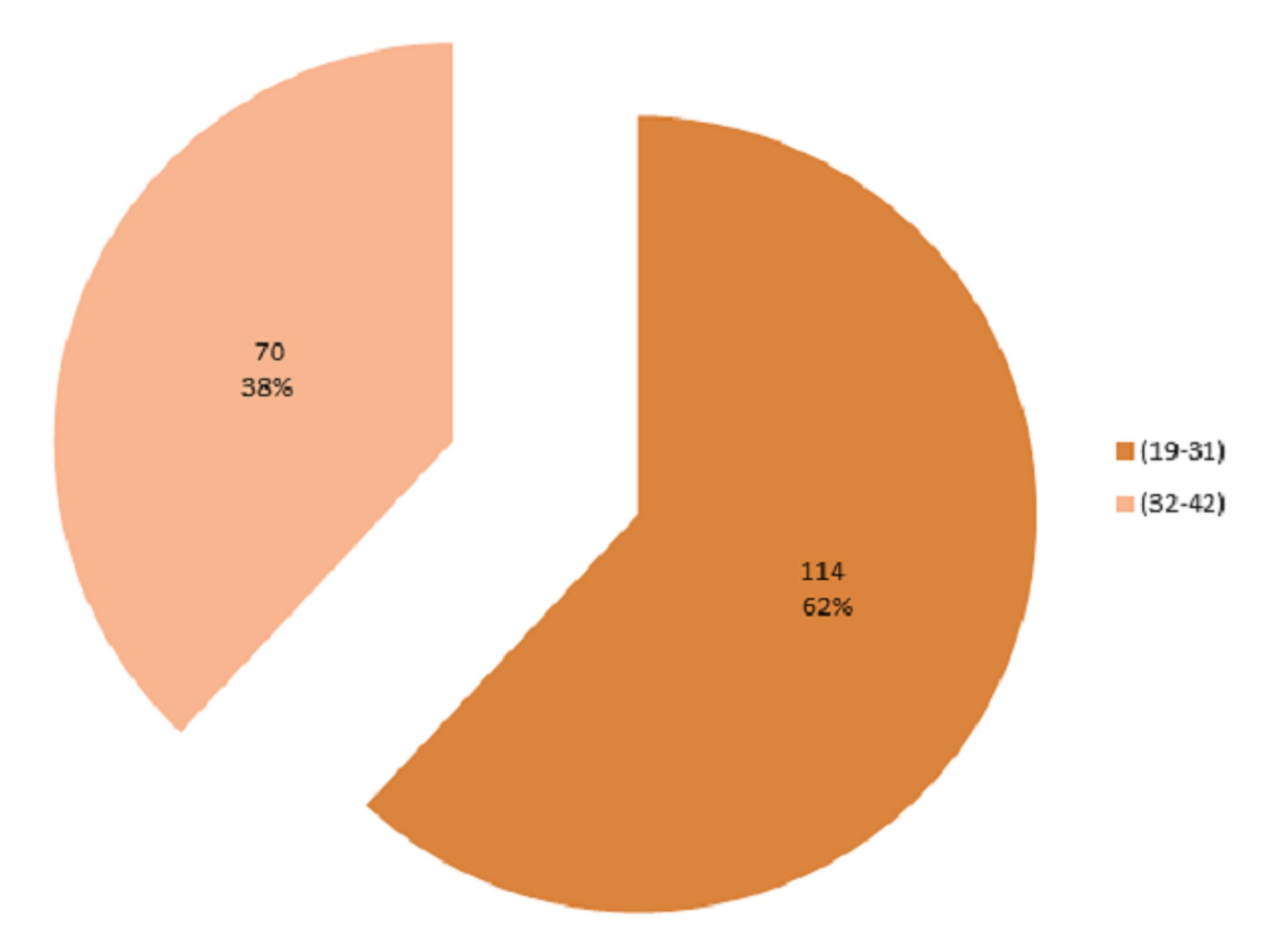
Percentage of patients according to age groups

**Figure 3:**
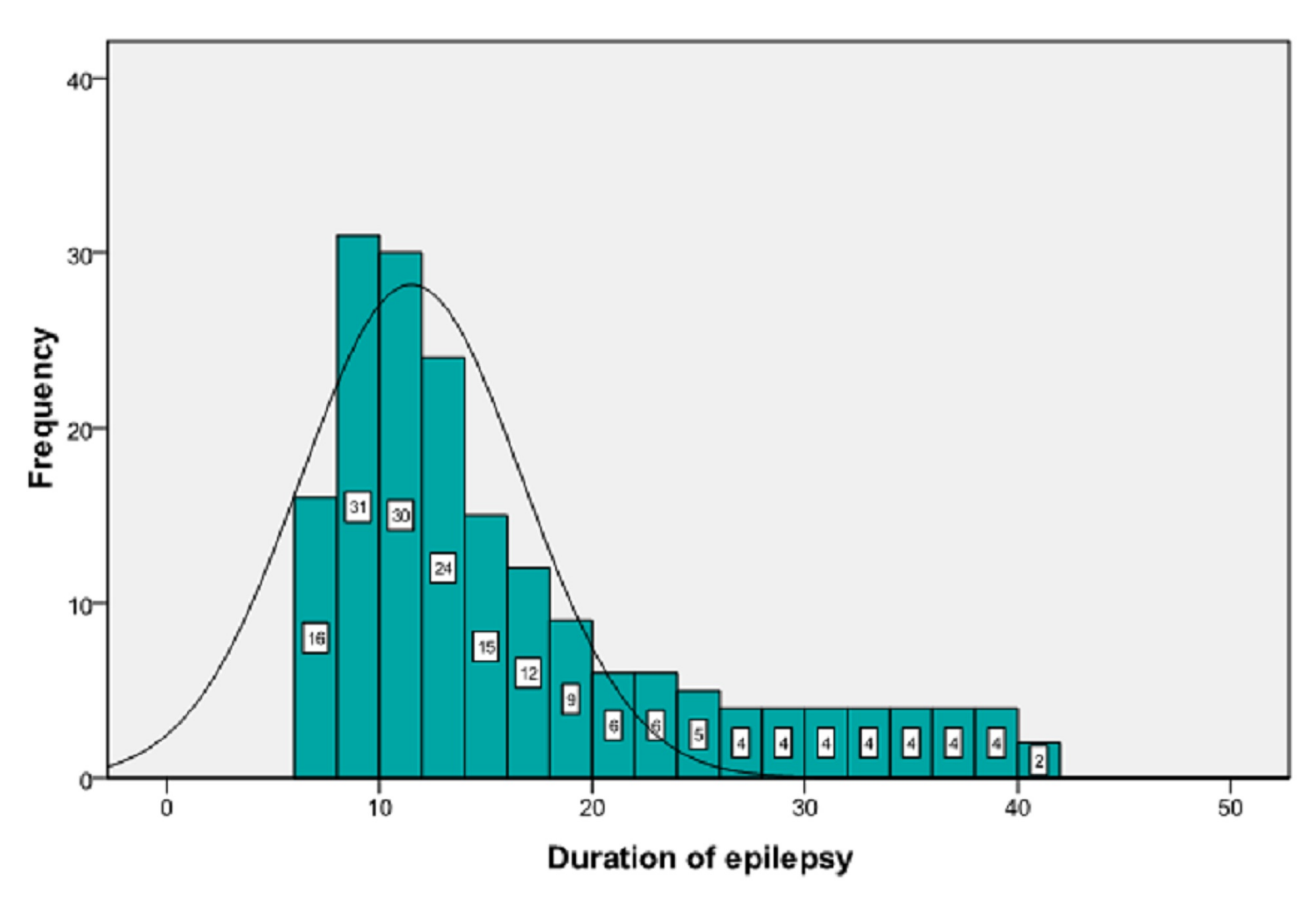
Frequency distribution of epilepsy duration

**Figure 4:**
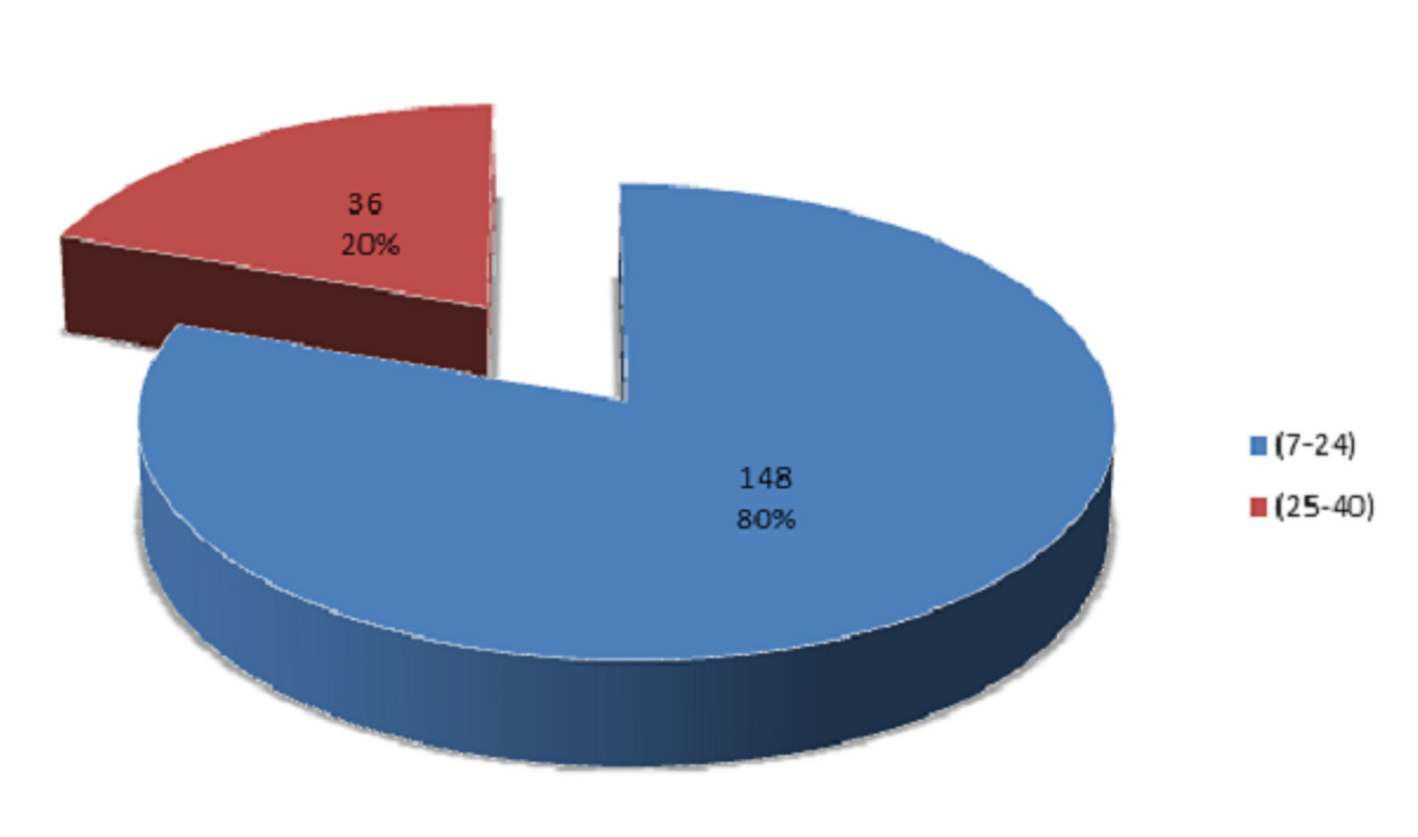
Percentage of patients according to the duration of epilepsy groups

**Figure 5:**
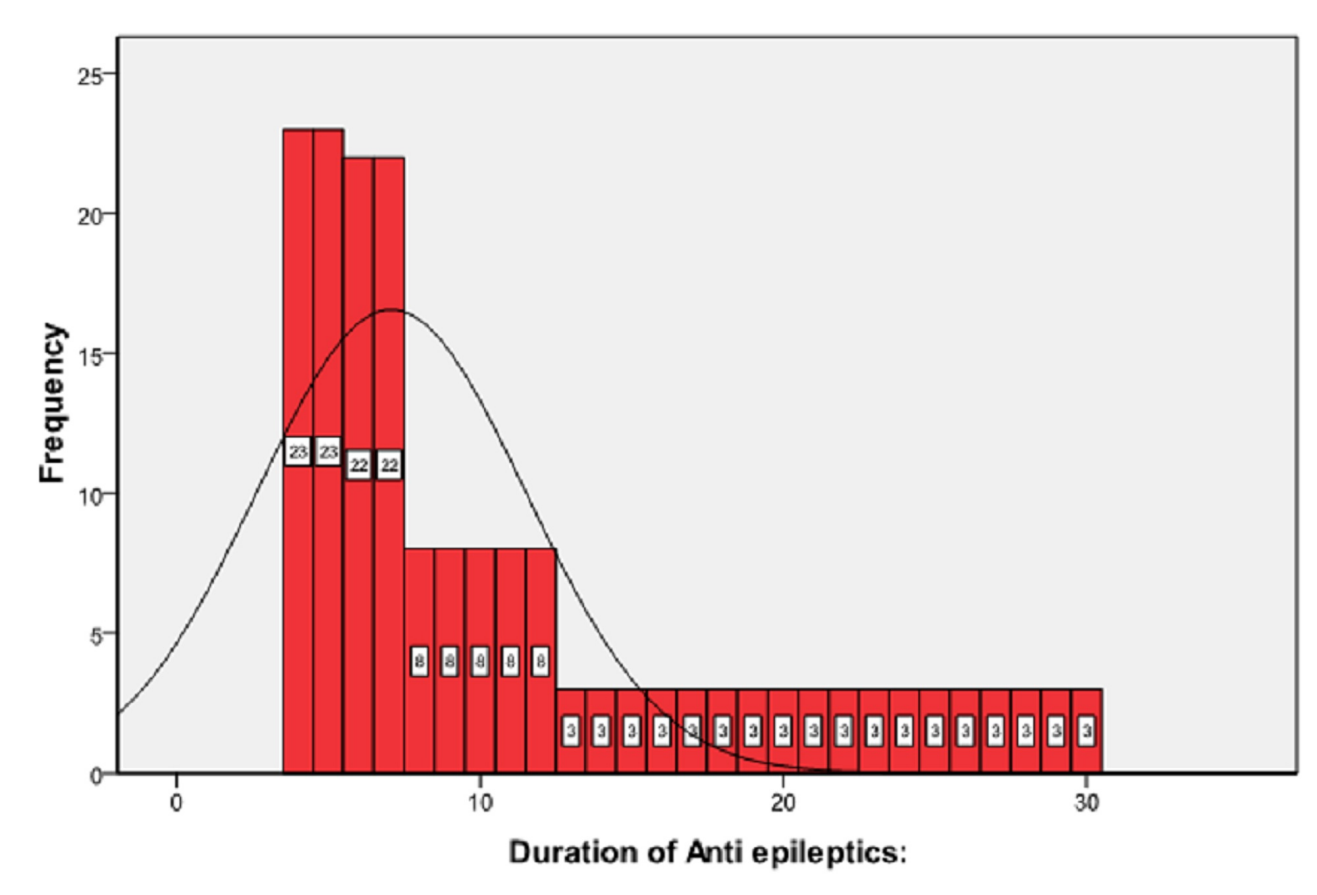
Frequency distribution of anti-epileptics duration

A total of 184 female patients of reproductive age were selected for the study. The mean age was 29.92 ± 4.67 years. The distribution of age is presented in Figure 1. The age was stratified into two groups. The frequency and percentages are presented in Figure 2. In our study, the mean duration of epilepsy was 15.96 ± 8.85 months. The distribution of the duration of epilepsy is presented in Figure 3. The duration of epilepsy was stratified into two groups. The frequency and percentages are presented in Figure 4. The mean duration of antiepileptic drugs was 11.16 ± 7.53 months. The distribution of the duration of antiepileptic drugs is presented in Figure 5. 

**Figure 6:**
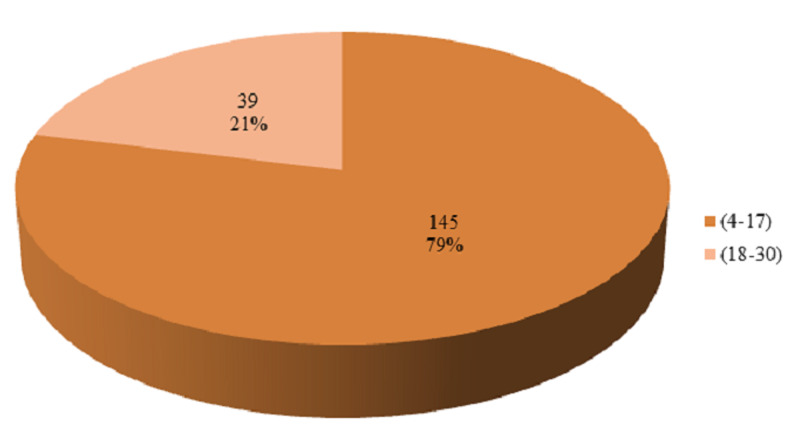
Percentage of patients according to the duration of antiepileptic groups

The duration of antiepileptic drugs was stratified into two groups. The frequency and percentages are presented in Figure 6. In our study, the most common anti-epileptic drug used was levetiracetam, which was used in 108 patients (58.7%). Carbamazepine was used in 63 patients (34.2%) and phenytoin in 13 patients (7.1%). In 73 patients (39.7%) EEG showed increased seizure activity during particular phases of the menstrual cycle. Catamenial epilepsy was seen in 73 patients (39.7%). The distribution of catamenial epilepsy is presented in Table 1. The frequencies of age groups, duration of epilepsy, duration of antiepileptics, anti-epileptic drugs were calculated according to catamenial epilepsy. The results are presented in Tables 2-5. The stratification according to age, duration of epilepsy, duration of antiepileptics, anti-epileptic drugs was done to observe the effect of these modifiers on catamenial epilepsy.

**Table 1:** Frequency distribution of catamenial epilepsy (n=184)

Catamenial epilepsy	Frequency (n)	Percentage (%)
No	111	60.3%
Yes	73	39.7%
Total	184	100%

**Table 2:** Catamenial epilepsy according to age (n=184) ' Not significant at 0.05 level

Age	Catamenial epilepsy: no	Catamenial epilepsy: yes	Total		P-value	
19-31 years	67 (36.41%)	47 (25.85%)	114 (62.7%)		0.386'	
32-42 years	44 (23.91%)	26 (14.2%)	70 (38.3%)			
Total	111 (60.3%)	73 (39.7%)	184 (100%)			

**Table 3:** Catamenial epilepsy according to the duration of epilepsy (n=184) ' Not significant at 0.05 level

Duration of epilepsy	Catamenial epilepsy: no	Catamenial epilepsy: yes	Total		P-value
7-24 months	79 (42.93%)	69 (37.5%)	148 (80.43%)		0.182'
25-40 months	32 (17.39%)	4 (2.17%)	36 (19.57%)		
Total	111 (60.3%)	73 (39.7%)	184 (100%)		

**Table 4:** Catamenial epilepsy according to the duration of antiepileptics (n=184)

Duration of antiepileptics	Catamenial epilepsy: no	Catamenial epilepsy: yes	Total		P-value
4-17 months	78 (42.39%)	67 (36.41%)	145 (78.8%)		0.016
18-30 months	33 (17.93%)	6 (3.26%)	39 (21.2%)		
Total	111 (60.3%)	73 (39.7%)	184 (100%)		

**Table 5:** Catamenial epilepsy according to antiepileptic drugs (n=184)

Antiepileptic drug	Catamenial epilepsy: no	Catamenial epilepsy: yes	Total	P-value
Carbamazepine	43 (23.4%)	20 (10.9%)	63 (34.2%)	0.000
Levetiracetam	68 (37%)	40 (21.7%)	108 (58.7%)	
Phenytoin	0	13 (7.1%)	13 (7.1%)	
Total	111 (60.3%)	73 (39.7%)	184 (100%)	

## Discussion

Catamenial epilepsy is categorized into various types, namely perimenstrual (ovulatory), periovulatory (ovulatory), or luteal (anovulatory cycle), in lieu of severity of seizures at different phases of the menstrual cycle. Women with frequent seizure episodes during any of the three menstruation phases must be diagnosed with catamenial epilepsy. A glimpse in the patient's diary in order to track seizure occurrence unveiled an increased incidence of seizures in ovulatory and perimenstrual phases; thus, the probability of catamenial epilepsy must be considered. As proven by multiple studies, catamenial epilepsy is not receptive to antiepileptics solely. In recent times, no definitive cure for catamenial epilepsy is discovered. However, studies are suggestive of hormonal and non-hormonal therapies [17]. Acetazolamide, a carbonic-anhydrase inhibitor, as a non-hormonal treatment, displayed its efficacy against catamenial epilepsy, but its efficacy declined with time, and resistance against it developed. Correspondingly, treatment with another drug, clonazepam, an intermediately acting benzodiazepine, showed a decline in the incidence of seizures in catamenial epilepsy. Nonetheless, prolonged consumption of clonazepam may lead to dependency and tolerability. Multiple studies documented the trial of either oral cyclical progesterone or intramuscular medroxyprogesterone injections efficacious in reducing and remission of seizures. High doses of oral progesterone (600mg/day) are given from day 16-25 of the menstrual cycle while opting for cyclical progesterone as treatment, and it is proved as a potential choice of treatment during the anovulatory phase. Three doses of intramuscular Depo-Provera®, twice-weekly 250, 250, and 150 mg, led to remission of seizures in a span of four months, as reported by Zimmerman et al. [18]. Nevertheless, seizures reoccurred, but the frequency was diminished. It is difficult to suggest an unmarried female opt for contraception or change their mentality regarding contraception as an anti-fertility drug. Najafi et al. reported similar mythologies in a study conducted on well-educated Malaysian women. The conclusions of their study reported the same misconceptions in interviewees regarding contraception and fertility [19].

In our study, the frequency of catamenial epilepsy is recorded as 39.7%, when compared to conclusions of other studies where extensive variability is reported in the prevalence of catamenial epilepsy. One study quoted about 10% to 70% of women being affected with epilepsy during menstruation. Subjected to data collection and definition utilized, the frequency of catamenial epilepsy ranges from 10%-78% [20]. Clinical evidence and animal models observed for epilepsy suggest excitatory effects of estrogen and inhibitory effects of progesterone on neuronal excitability and seizures [21]. The protective manifestations of progesterone against epilepsy in animal models are attributed to reducing the concentration of progesterone metabolite tetrahydroprogesterone (THP), also called allopregnanolone, a GABAA receptor modulating neurosteroid with anticonvulsant properties. The presence of increased production of progesterone as an outcome of good seizure control. The family physicians' vital role in the case of chronic diseases is healing the sufferer and managing the disease [22]. Management of epilepsy is based not only on managing the disorder and abolishing the risks but also on reducing risks and sustaining a normal life, and performing daily activities. Our study suggests the physician's vigilant role with no hesitation in reevaluating the cure and revising the diagnosis if seizures are unresponsive to treatment. An instant reevaluation will help in betterment the quality of life of suffering individuals and abort unnecessary suffering. Diagnosing the category of seizure is a demanding effort for physicians, as they seldomly witness the patient's seizure. Unfortunately, the patient is in no state of giving a history of episode, so diagnosis is solely based on conditions described by eyewitnesses. An accurate diagnosis of the type of seizure is important for opting for a definitive course of medication.

## Conclusions

Catamenial epilepsy is common in females of reproductive age. The physician should evaluate patients when the seizures are refractory to the treatment. The females should manage a seizure diary, which will be beneficial in the management of epilepsy. In women with epilepsy, catamenial epilepsy should be considered in the diagnosis when the seizures are refractory to management. Still, no specific treatment is available for catamenial epilepsy; however, hormonal therapy and steroids are proposed to be used in these cases. Thus there is no appropriate treatment for catamenial seizures, and in the reproductive age of females, one is uncertain which treatment will affect the fertility, cardiovascular system, and bones of females.
